# Nestin-Cre Mice Are Affected by Hypopituitarism, Which Is Not Due to Significant Activity of the Transgene in the Pituitary Gland

**DOI:** 10.1371/journal.pone.0011443

**Published:** 2010-07-06

**Authors:** Christophe Galichet, Robin Lovell-Badge, Karine Rizzoti

**Affiliations:** Division of Stem Cell Biology and Developmental Genetics, MRC National Institute for Medical Research, London, United Kingdom; Tufts University, United States of America

## Abstract

Nestin-Cre mice express Cre recombinase under control of the rat *nestin* promoter and central nervous system (CNS) enhancer. While endogenous Nestin is expressed in some other tissues including the pituitary gland, Nestin-Cre mice induce recombination predominantly in the CNS. For this reason, they have been widely used to explore gene function or cell fate in the latter. Pituitary hormonal deficiencies, or hypopituitarism, are associated with a wide range of symptoms and with a significant morbidity. These can have a neural and/or a pituitary origin as the gland's secretions are controlled by the hypothalamus. We report here that Nestin-Cre mice themselves are affected by mild hypopituitarism. Hence, physiological consequences are expected, especially in combination with defects resulting from Cre mediated deletion of any gene under investigation. To further investigate the origin of this phenotype, we re-examined the activity of the transgene. We compared it with expression of Nestin itself in the context of the hypothalamo-pituitary axis, especially in the light of a recent report showing pituitary Nestin-Cre activity, which contrasts with previous data. Our results disagree with those of this recent study and do not support the claim that Nestin positive cells are present in the pituitary anlagen, the Rathke's pouch (RP). Moreover we did not observe any significant activity in the post-natal pituitary, in agreement with the initial report.

## Introduction

Disorders affecting the hypothalamo-pituitary axis can result in single or multiple hormonal deficiencies, or hypopituitarism. They are associated with a wide range of symptoms, reflecting the roles of the different pituitary hormones, and with a significant morbidity. They can result from pituitary or/and central nervous system (CNS) defects as the gland's secretions are under hypothalamic control. Isolated Growth Hormone Deficiency (IGHD) is prevalent, with an incidence of 1 in 4000–10000 live births [1]. Some of these disorders have been shown to stem from abnormal development of the hypothalamo-pituitary axis, and several genes responsible for these defects have been characterized. However, because of the intricate interactions between both components of the axis, the hypothalamus within the CNS and the pituitary gland, which mostly originates from oral ectoderm, and also the complex expression pattern of some of the genes whose deletion or mutation results in hypopituitarism, it has sometimes been difficult to pinpoint the origin of defects ultimately affecting post-natal pituitary secretions (for review see [2]).

In order to investigate complex phenotypes, conditional deletion of a gene of interest is often required. To achieve this goal the Cre-Lox system has been widely used. The Cre enzyme from the bacteriophage P1 catalyzes DNA recombination between LoxP sites allowing, in particular, deletion of the intervening DNA fragment [3]. Deletion can be controlled both in time and space by using a tissue specific promoter to drive Cre expression. Nestin-Cre mice, expressing Cre recombinase under control of the rat *nestin* promoter and its CNS specific enhancer, were initially generated to specifically inactivate the glucocorticoid receptor (*Gr*) gene in the nervous system [4]. Indeed, while endogenous Nestin is expressed in the post-natal pituitary [5], the CNS specific transgene does not delete *Gr* in the gland [4]. Since then, Nestin-Cre mice have been widely used to investigate gene function or cell fate in the CNS. In the context of the hypothalamo-pituitary axis, they provide a useful tool in order to distinguish whether a phenotype originates from a pituitary or a hypothalamic defect, when the protein of interest is expressed in both tissues, during embryonic development and/or in the adult. In recent years however, Cre activity or transgene insertion, have sometimes been associated with side effects. We report here that Nestin-Cre mice are themselves affected by hypopituitarism and this is expected to have physiological consequences.

Moreover, in contrast with the initial study reporting Nestin-Cre generation [4], Nestin-Cre activity was recently reported in the pituitary [6]. Cell lineage analysis using this transgene, and an inducible version of it (Nestin-Cre-ER), suggested that Nestin positive cells represent adult stem cells. As this would have implications for using Nestin-Cre in the context of the hypothalamo-pituitary axis to distinguish a pituitary from a CNS phenotype, we decided to investigate this further. We therefore characterized Nestin-Cre activity and Nestin expression itself in the context of the hypothalamo-pituitary axis both in the embryo and in the adult. Our results differ from those of Gleiberman *et al.* and do not support the claim that Nestin positive cells are present in the early embryonic pituitary. Moreover we, and others [4], [7], do not detect any significant activity of this transgene in the adult pituitary.

## Results

### Nestin-Cre mice display mild hypopituitarism

**Figure 1:**
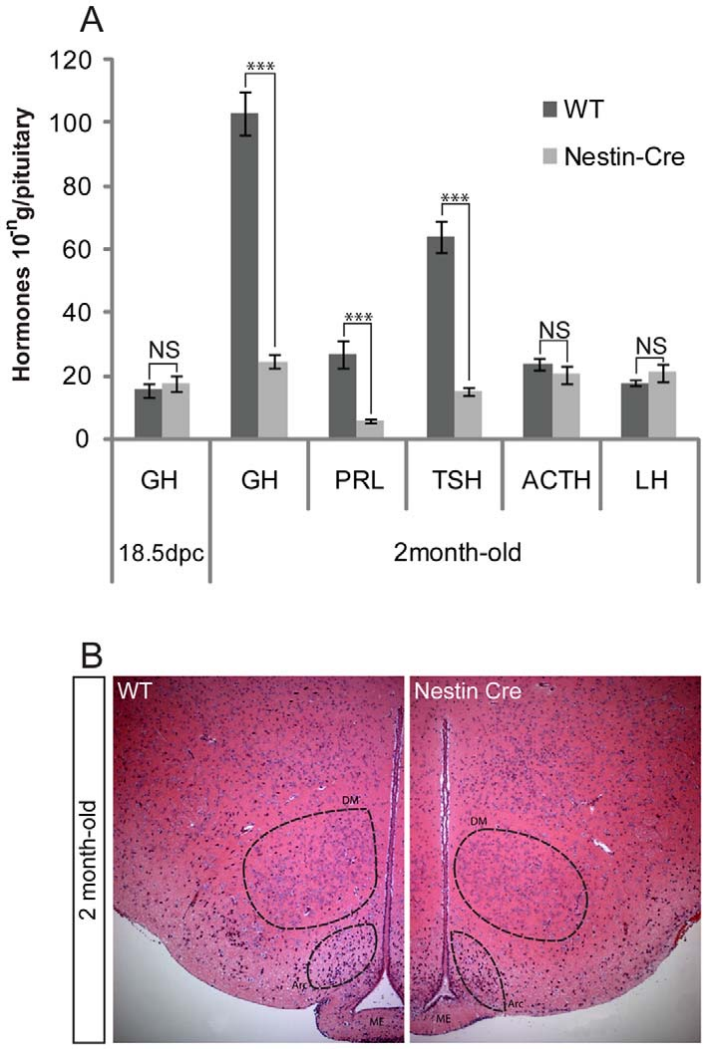
Anterior pituitary contents and hypothalamus morphology. (A)Graphic representation of anterior pituitary hormonal contents measured by RIA of 18.5dpc and 2 month-old wild-type and Nestin-Cre samples (five samples/genotype were examined). Hormonal contents correspond to 10^−8^ g/pituitary for GH at 18.5dpc (values: WT: 15.28 SEM±2.2 Nestin-Cre: 17.1 SEM±2.4) and 10^−6^ g/pituitary for GH (values: WT: 103.3 SEM±6.94 Nestin-Cre: 24.37 SEM±2.34), 10^−7^ for PRL (values: WT: 26.67 SEM±4.49 Nestin-Cre: 5.57 SEM±0.76) and LH (values: WT: 17.5 SEM±0.98 Nestin-Cre: 21 SEM±2.86) and 10^−8^ for TSH (values: WT: 63.9 SEM±4.93 Nestin-Cre: 32.57 SEM±1.19) and ACTH (values: WT: 23.73 SEM±1.82 Nestin Cre: 20.24 SEM±2.91) in 2 month-old animals; ***p<0.001; NS: not significant. (B) Hematoxylin and eosin stained transverse sections through the hypothalamus of 2 month-old wild-type and Nestin-Cre males. ME: Median eminence; Arc: Arcuate nucleus; DM: Dorsomedial hypothalamic nucleus.

Anterior pituitary hormone contents were measured by radio-immunoassay (RIA) just before birth (18.5dpc), when pituitary hormones are present but their secretion is not yet under hypothalamic control, and in 2 month-old adults, comparing heterozygous Nestin-Cre and controls (littermates or wild-type animals from the same genetic background). Before birth, Growth Hormone (GH) contents are similar in both groups (Figure 1A). However, in adult, Nestin-Cre mice display a 70% to 80% reduction in GH, prolactin (PRL) and thyroid stimulating hormone (TSH) contents while luteinizing hormone (LH) and adrenocorticotropic hormone (ACTH) levels are not affected (Figure 1A). We obtained the same results in males and females (data not shown). To exclude the possibility that our colony is affected by an additional mutation leading to the phenotype, we confirmed these results on animals bred in another colony (data not shown). In contrast, mice homozygous for β-actin-Cre [8], which express Cre ubiquitously, were not affected by hypopituitarism (data not shown). This implies either that relative levels of Cre activity are critical or that the Nestin-Cre transgene has compromised the activity of a gene at its integration site. Rather than a defect within the pituitary itself, the phenotype is more consistent with compromised hypothalamic development or function because the deficits only start post-natally, when the secretions are under CNS control. Moreover, deficiencies are specific to a subset of endocrine cells, those secreting GH, PRL and TSH, which belong to the same lineage defined by expression of the transcription factor Pit-1. This factor is expressed before differentiation of these endocrine cells in the embryo and is required for their post-natal expansion (for review see [2]). This suggests that the hypothalamic input on Pit-1 positive cells to control hormonal secretion, synthesis or cell proliferation, is affected in these mice. Histological examination of different hypothalamic nuclei (arcuate, dorso and ventro-medial, paraventricular, medial and supra-mammilary) in Nestin-Cre animals did not however reveal any gross morphological defect (Figure 1B).

### The Nestin-Cre transgene does not show any significant activity in the pituitary gland

In the context of the hypothalamo-pituitary axis, Nestin itself is known to be expressed in embryonic and adult neural stem/progenitor cells. In the post-natal gland, Nestin is found in different types of non-endocrine cells [5]. A subset of Nestin^+ve^ cells comprise folliculo-stellate (FS) cells, a heterogenous population whose function is unclear. Nestin is also present in the periluminal cell layer, which is proposed to contain progenitors. Finally other Nestin^+ve^ cells are part of the gland's connective tissue [5]. In contrast, Nestin-Cre expression was initially reported to be CNS specific [4]. However, a recent report challenged this observation by showing that a Nestin-GFP transgene generated using similar regulatory elements to that of the original Nestin-Cre transgene is expressed in the pituitary anlagen or Rathke's pouch (RP) [6]. Moreover Nestin-Cre and an inducible Nestin-Cre-ER version were shown to generate significant staining in the adult gland, in cell lineage tracing experiments [6]. This had not been reported previously. To investigate this further, we re-evaluated the recombination pattern given by Nestin-Cre in the context of hypothalamo-pituitary axis development, by making use of the Rosa26reporterEYFP allele. This gives EYFP expression in cells in which the transgene is active and in all their descendants. We compared this pattern with endogenous Nestin expression.

**Figure 2:**
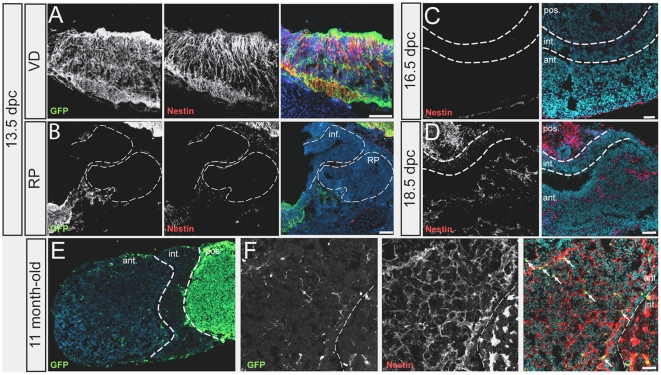
Nestin-Cre activity and Nestin expression. (A, B) Immunofluorescence for GFP and Nestin in 13.5dpc NestinCre; R26RYFP embryo. Ventral diencephalon (VD) (A) and Ratkhe's pouch (RP) (B). Note that EYFP expression always correlates with that of Nestin within the CNS. (C, D) Immunofluorescence for Nestin on 16.5dpc (C) and 18.5dpc (D) wild-type pituitaries. (E, F) Immunofluorescence for GFP (E) and for GFP and Nestin (F) on Nestin-Cre; R26ReporterEYFP eleven month-old pituitaries. The arrows point to Nestin^+ve^, EYFP^+ve^ cells. Scale bar: 50 µm; ant.: anterior; int.: intermediate; pos.: posterior; inf: infundibulum

In the embryo (13.5dpc), Nestin co-localizes with EYFP in the ventral diencephalon (VD), but both are absent from the infundibulum, a specific domain within the VD that gives rise to the pituitary stalk and posterior lobe of the gland, and also from the RP (Figure 2A,B). In fact, we did not detect any pituitary Nestin expression or Nestin-Cre activity up to 16.5dpc, although at 18.5dpc Nestin protein was present in the developing gland (Figure 2C,D and data not shown). This is in agreement with the results of Imai *et al.* who found lack of recombination in the developing pituitary at 15.5dpc with a Nestin-Cre strain independently generated with the rat *Nestin* promoter and CNS enhancer [9]. Therefore, in the embryo, until at least 16.5dpc, Nestin-Cre and Nestin itself display the same pattern in the context of the developing hypothalamo-pituitary axis, both being absent from the embryonic pituitary. This is in contrast with the observation of Gleiberman's *et al.* showing Nestin-GFP expression in RP [6].

Post-natally, Nestin and EYFP are both expressed in the adult posterior pituitary, which is of neural origin (Figure 2E, F). However, very few EYFP^+ve^ cells result from Nestin-Cre activity in the adult anterior pituitary (Figure 2E, EYFP^+ve^ cells represent 0.7% of anterior lobe cells in this 11 month-old specimen; 8547 DAPI^+ve^ nuclei were counted). Moreover, these are also Nestin^+ve^ and therefore represent only a minute fraction of the Nestin^+ve^ population (in contrast, the Rosa26reporterEYFP allele recombined ubiquitously in the pituitary gland when bred to β-actin-Cre; data not shown). This result agrees with the initial study describing Nestin-Cre mice [4] and also with a more recent study, using the same strain and describing Cre reporter expression in the posterior lobe but not in the anterior pituitary [7]. However, these results conflict with the Nestin-GFP transgene expression observed in the adult in Gleiberman's *et al.* This discrepancy may reflect the use of not quite identical (although they are very similar) regulatory elements or different integration sites of the transgenes. Moreover, our results also contrast with the lineage tracing data of Gleiberman *et al.* where, using the same Nestin-Cre, GFP reporter expression was found in many pituitary cells (up to 20% of the anterior lobe in 5 month-old animals) including endocrine cells. It should be noted that different reporter mice were used in this study [10] and in Gleiberman *et al*. [11]. While in both cases the same locus (Rosa26) was targeted, the constructs are slightly different.

## Discussion

Nestin-Cre mice are widely used to dissect gene and cell function in the CNS. We report here that these mice are affected by mild hypopituitarism and this is expected to have physiological consequences, especially in combination with any defects resulting from loss of the gene under investigation. The use of Nestin-Cre control samples is therefore necessary to distinguish real consequences of deletion of the gene under investigation from side effects resulting from Nestin-Cre associated hypopituitarism. In the context of the hypothalamo-pituitary axis, their specific CNS activity was recently challenged by a study reporting activity of this, and another similar transgene (Nestin-GFP) both in the embryonic and adult pituitary [6]. Based on cell lineage analysis, this report suggested that Nestin positive cells represent adult pituitary stem cells. In contrast, we, and others, did not detect any Nestin expression or Nestin-Cre activity [9] until at least 16.5dpc. In the adult, very few cells showed transgene activity, in agreement with previous studies [4], [7]. Therefore our data do not support the claim that Nestin positive cells are present in the early pituitary and cell lineage analysis using Nestin-Cre did not generate any significant labelling in the post-natal anterior pituitary.

### Nestin-Cre mice and hypopituitarism

There have now been several reports of side effects and pitfalls associated with Cre activity or transgene insertion. These include apoptosis and anaemia in embryonic development, impairment of pancreatic function (glucose intolerance), microcephaly and hydrocephaly, and chromosome rearrangements in spermatids [12], [13], [14], [15]. The mammalian genome contains cryptic or “pseudo-LoxP” sites that can be used as functional recognition sites for the enzyme [16], [17]. It has therefore been hypothesized that high expression or long exposure to Cre activity might trigger recombination leading to chromosomal rearrangements in mammalian cells *in vivo*. Transgene integration site might also affect activity of surrounding genes, leading to defects. Here, we have not analyzed the origin of the hormonal deficiencies. However, β-actin-Cre animals, that express Cre ubiquitously [8], were not affected by hypopituitarism suggesting that either relative levels of Cre activity are critical or that the site of insertion of the transgene might be responsible for the defect in Nestin-Cre mice.

The hypopituitarism displayed by Nestin-Cre mice is post-natal and specific to the Pit-1 lineage (GH, PRL and TSH cells). As we have shown here that the transgene is active in the CNS but not in the embryonic pituitary and at very low level in the adult gland, a defect within these endocrine cells, or affecting their emergence in the embryo is very unlikely to be at the origin of the phenotype. The embryonic VD is important early on for RP morphogenesis and maintenance and Nestin-Cre is active in this tissue. However, later on, as pituitary endocrine cells differentiate, VD does not seem to have a significant effect on the developing gland. Indeed, up to now, mutations affecting VD therefore hypothalamus development (*Gsh-1* mutants [18]) or function (*little* mouse, *GFR receptor* mutants [19]) have only been shown to affect the post-natal pituitary whose morphogenesis was normal. In Nestin-Cre mice, it is therefore rather the post-natal hypothalamus, controlling the gland's secretions, that is likely to be responsible for the hypopituitarism. The hypothalamus modulates hormonal secretion, synthesis and endocrine cell proliferation. Any of these inputs directed specifically to the endocrine cells of the Pit-1 lineage could be affected in the Nestin-Cre mice inducing as a consequence a reduction in pituitary hormonal contents. Non-specific Cre activity within these regulatory cells or integration of the transgene in a site important for their differentiation or function may underline the defect. This defect is expected to be rather subtle, as histological examination of the hypothalamus did not reveal any gross morphological abnormality.

Finally, the hypopituitarism displayed by Nestin-Cre mice has implications for using them to study pituitary phenotypes. Where Nestin-Cre is used, pituitary contents in conditional mutants must be compared with that of Nestin-Cre animals without the floxed gene as controls. More generally, mild hypopituitarism would be compensated for in young animals by increasing hypothalamic input, but, as compensation eventually fails in the adult, deficiencies would slowly develop with potential physiological consequences (e.g. IGF1 deficiency, I.C. Robinson, personal communication). In particular it has been shown that adult onset GH and IGF-1 deficiencies affect neurogenesis [20]. These effects would arise in combination with any defects resulting from loss of the gene under study.

### The Nestin-Cre transgene is not significantly active in the pituitary

Nestin expression in the pituitary had only been described post-natally to our knowledge. It is present in different non-endocrine cells [5], in a subset of FS cells whose function remains unclear, in potential progenitors located in the epithelium flanking the pituitary cleft and in supporting cells (for review see, [21]). Recently, we have shown that spheres obtained from adult pituitaries [22] express SOX2, can proliferate and also differentiate, suggesting that SOX2 positive cells may represent adult pituitary progenitor/stem cells [23]. In the spheres, Nestin expression was initiated after SOX2 suggesting that Nestin positive cells may represent more determined cells, corresponding to transit amplifying progenitors. Therefore, there is clearly a link between Nestin expression and adult progenitor/stem cells in the pituitary.

We first analysed Nestin expression in the embryo and did not see any expression before 18.5 dpc. In parallel, we, and others [9], did not detect any Nestin-Cre activity until at least 16.5dpc. In contrast, Gleiberman *et al.* report expression of a Nestin-GFP transgene in RP at 11.5dpc. This discrepancy may reflect use of slightly different promoter/enhancer elements, as Gleiberman *et al.* derived their Nestin-GFP transgene independently, although from similar sequences, or it might reflect different integration sites. However, if Nestin itself is not expressed in RP, the transgene activity is not reflecting expression of the protein at this stage and location and is therefore likely to be ectopic.

In the adult, when Nestin is now present, we show that Nestin-Cre activity represents only a minute fraction of Nestin-positive cells (0.7% of total anterior lobe cells), in agreement with others [4], [7]. This is also consistent with a lack of significant activity of the transgene in the embryo. However, these results conflict with the lineage tracing data of Gleiberman *et al.*, where GFP reporter expression is found in many pituitary cells (up to 20% of the anterior lobe) including endocrine cells. The fact that these differentiated endocrine cells are GFP^+ve^ prompted these authors to suggest that the hormone negative, Nestin^+ve^, and by extension Nestin-Cre^+ve^ cells represent progenitors. In our hands, the few EYFP^+ve^ cells we have found in the adult are unlikely to represent progenitors as they always co-expressed Nestin. They may therefore belong to another sub-population of Nestin^+ve^ cells. The discrepancy observed in activity of the transgene in the pituitary is difficult to explain as the mice used here, elsewhere [7] and in Gleiberman's study have the same origin ([4], The Jacskon laboratory). As described above, the reporter mice used here and in Gleiberman *et al.* are not identical. They both result from a targeted insertion in the Rosa26 locus but the constructs are slightly different. However, in a study where the two reporter lines were compared, it was concluded that the one we used is the most efficient, so this does not provide an explanation for the discrepancy [24]. Moreover, expression of the *Gr* gene was intact in the gland in the initial study [4] and, using a Rosa26LacZ reporter, Wettschureck *et al*. also failed to observe any anterior pituitary expression [7]. As we discussed above, at least some Nestin positive cells are most probably adult pituitary progenitors. Therefore the transgene used in Gleiberman *et al.* could display genuine expression in the adult, reflecting Nestin expression. However ectopic activity in the embryonic gland such as the one observed with the Nestin-GFP transgene in RP progenitors at 11.5dpc [7] would give rise to significant staining of endocrine cells post-natally, as anterior pituitary cells derive from RP (for review see [2]). To identify Nestin positive cells as stem cells would require showing exclusive co-localization of Cre and Nestin.

In conclusion, our work highlights the importance of carrying out controls both to relate the activity of an enhancer driving Cre transgene expression to that of the endogenous gene, and to reveal whether the Cre transgene leads to a relevant phenotype by itself.

## Materials and Methods

### Ethics Statement

All experiments carried out on mice were approved under the UK Animal (scientific procedures) Act (Project licence 80/1949).

### Mice

We maintain Nestin-Cre [4] and β-actin-Cre mice on an MF1 background (random bred, NIMR). Nestin-Cre mice were purchased from the Jackson Laboratory (Origin, R. Klein, EMBL). R26REYPF mice were obtained from S. Srinivas [10]. Embryos were obtained by mating homozygous Rosa26reporterEYFP females to heterozygous Nestin-Cre males. The day of vaginal plug corresponds to 0.5dpc. For RIA, we analyzed heterozygous Nestin-Cre animals and homozygous β-actin-Cre mice. Age-matched MF1 mice and Cre-negative siblings were used as controls. Nestin-Cre mice (Origin, R. Klein, EMBL) maintained on C57BL6 background were obtained from the London Research Institute (LRI, CRUK) and used as an independent colony to confirm the hypopituitarism phenotype.

### Immunofluorescence and histology

For frozen sections, embryos were fixed in 4% paraformaldehyde for 1 h at 4°C, while adults were perfused intracardially with 4% paraformaldehyde. Samples were then cryoprotected in 30% sucrose and embedded in OCT (BDH). Immunufluorescence was performed on 12 µm sections using a 1∶500 dilution of a rabbit antibody to GFP (recognizing EYFP – Chemicon) and a 1∶100 dilution of a mouse antibody to Nestin (DSHB, USA). Detection was performed by using a 1∶500 dilution of anti-rabbit and anti-mouse antibodies conjugated to Alexa-488 and Alexa-555 respectively (Molecular Probes). For histology, 2 month-old brains were fixed in Bouin's solution overnight, dehydrated and embedded in paraffin. Samples were then sectioned and stained with hematoxylin and eosin.

### Radioimmunoassay

Pituitaries were homogenised in phosphate-buffer saline and assayed for anterior pituitary hormones using National Hormone and Pituitary Program reagents kindly provided by A.L. Parlow, as described in [25].

## References

[1] Alatzoglou KS, Dattani MT (2009). Genetic forms of hypopituitarism and their manifestation in the neonatal period.. Early Hum Dev.

[2] Kelberman D, Rizzoti K, Lovell-Badge R, Robinson IC, Dattani MT (2009). Genetic regulation of pituitary gland development in human and mouse.. Endocr Rev.

[3] Sauer B, Henderson N (1989). Cre-stimulated recombination at loxP-containing DNA sequences placed into the mammalian genome.. Nucleic Acids Res.

[4] Tronche F, Kellendonk C, Kretz O, Gass P, Anlag K (1999). Disruption of the glucocorticoid receptor gene in the nervous system results in reduced anxiety.. Nat Genet.

[5] Krylyshkina O, Chen J, Mebis L, Denef C, Vankelecom H (2005). Nestin-immunoreactive cells in rat pituitary are neither hormonal nor typical folliculo-stellate cells.. Endocrinology.

[6] Gleiberman AS, Michurina T, Encinas JM, Roig JL, Krasnov P (2008). Genetic approaches identify adult pituitary stem cells.. Proc Natl Acad Sci U S A.

[7] Wettschureck N, Moers A, Wallenwein B, Parlow AF, Maser-Gluth C (2005). Loss of Gq/11 family G proteins in the nervous system causes pituitary somatotroph hypoplasia and dwarfism in mice.. Mol Cell Biol.

[8] Lewandoski M, Meyers EN, Martin GR (1997). Analysis of Fgf8 gene function in vertebrate development.. Cold Spring Harb Symp Quant Biol.

[9] Imai F, Hirai S, Akimoto K, Koyama H, Miyata T (2006). Inactivation of aPKClambda results in the loss of adherens junctions in neuroepithelial cells without affecting neurogenesis in mouse neocortex.. Development.

[10] Srinivas S, Watanabe T, Lin CS, William CM, Tanabe Y (2001). Cre reporter strains produced by targeted insertion of EYFP and ECFP into the ROSA26 locus.. BMC Dev Biol.

[11] Mao X, Fujiwara Y, Chapdelaine A, Yang H, Orkin SH (2001). Activation of EGFP expression by Cre-mediated excision in a new ROSA26 reporter mouse strain.. Blood.

[12] Lee JY, Ristow M, Lin X, White MF, Magnuson MA (2006). RIP-Cre revisited, evidence for impairments of pancreatic beta-cell function.. J Biol Chem.

[13] Naiche LA, Papaioannou VE (2007). Cre activity causes widespread apoptosis and lethal anemia during embryonic development.. Genesis.

[14] Forni PE, Scuoppo C, Imayoshi I, Taulli R, Dastru W (2006). High levels of Cre expression in neuronal progenitors cause defects in brain development leading to microencephaly and hydrocephaly.. J Neurosci.

[15] Schmidt EE, Taylor DS, Prigge JR, Barnett S, Capecchi MR (2000). Illegitimate Cre-dependent chromosome rearrangements in transgenic mouse spermatids.. Proc Natl Acad Sci U S A.

[16] Higashi AY, Ikawa T, Muramatsu M, Economides AN, Niwa A (2009). Direct hematological toxicity and illegitimate chromosomal recombination caused by the systemic activation of CreERT2.. J Immunol.

[17] Thyagarajan B, Guimaraes MJ, Groth AC, Calos MP (2000). Mammalian genomes contain active recombinase recognition sites.. Gene.

[18] Li H, Zeitler PS, Valerius MT, Small K, Potter SS (1996). Gsh-1, an orphan Hox gene, is required for normal pituitary development.. EMBO J.

[19] Lin SC, Lin CR, Gukovsky I, Lusis AJ, Sawchenko PE (1993). Molecular basis of the little mouse phenotype and implications for cell type-specific growth.. Nature.

[20] Lichtenwalner RJ, Forbes ME, Sonntag WE, Riddle DR (2006). Adult-onset deficiency in growth hormone and insulin-like growth factor-I decreases survival of dentate granule neurons: insights into the regulation of adult hippocampal neurogenesis.. J Neurosci Res.

[21] Vankelecom H (2007). Non-hormonal cell types in the pituitary candidating for stem cell.. Semin Cell Dev Biol.

[22] Chen J, Hersmus N, Van Duppen V, Caesens P, Denef C (2005). The adult pituitary contains a cell population displaying stem/progenitor cell and early embryonic characteristics.. Endocrinology.

[23] Fauquier T, Rizzoti K, Dattani M, Lovell-Badge R, Robinson IC (2008). SOX2-expressing progenitor cells generate all of the major cell types in the adult mouse pituitary gland.. Proc Natl Acad Sci U S A.

[24] Young KM, Mitsumori T, Pringle N, Grist M, Kessaris N (2010). An Fgfr3-iCreER(T2) transgenic mouse line for studies of neural stem cells and astrocytes.. Glia.

[25] McGuinness L, Magoulas C, Sesay AK, Mathers K, Carmignac D (2003). Autosomal dominant growth hormone deficiency disrupts secretory vesicles in vitro and in vivo in transgenic mice.. Endocrinology.

